# Clinical features and outcomes of neck lymphatic metastasis in ovarian epithelial carcinoma

**DOI:** 10.1186/1477-7819-11-255

**Published:** 2013-10-03

**Authors:** Chien-Wen Chen, Pao-Ling Torng, Chi-Ling Chen, Chi-An Chen

**Affiliations:** 1 Department of Obstetrics and Gynecology, National Taiwan University Hospital and National Taiwan University College of Medicine, No. 7, Chung Shan South Road, Taipei 10002, Taiwan; 2 Department of Health, Penghu Hospital, 10 Chung-Cheng Road, Makung, Penghu 880-41, Taiwan; 3 Graduate Institute of Clinical Medicine, National Taiwan University College of Medicine, No. 7, Chung Shan South Road, Taipei 10002, Taiwan

**Keywords:** Epithelial ovarian cancer, Neck lymph node metastasis, Survival

## Abstract

**Background:**

Neck lymph node metastasis (NLNM) in epithelial ovarian cancer (EOC) is rare and treated as advanced stage cancer. However, ovarian cancer with lymphatic metastasis may manifest a different clinical course from peritoneal carcinomatosis.

**Methods:**

The authors retrospectively assessed 20 patients with EOC and pathologically diagnosed as NLNM between January 2001 and December 2010. The patients were divided into two groups according to the time of NLNM identification. Statistical methods included Kaplan-Meier, log-rank, and Cox regression analysis.

**Results:**

Eleven patients were diagnosed with NLNM at the same time of surgical exploration of EOC (Group A) and nine patients at cancer recurrence 43.3 months after initial surgery (Group B). In Group A, patients with tumors confined to the pelvic cavity had no recurrence or had isolated lymph node recurrence (ILNR), and survived longer than patients with abdominal tumor spreading (*P* = 0.0007). In Group B, 2 patients showed ILNR. The median survival time after NLNM was 42 months in Group A and 6 months in Group B (*P* = 0.01). Cox model demonstrated that non-serous histology, brain metastasis, and NLNM identified at cancer recurrence were major predictors for poor overall survival (Hazard ratio [HR] = 18.67, 6.93, and 4.52; *P* = 0.01, 0.02, and 0.04, respectively).

**Conclusions:**

A subgroup of EOC patients with NLNM who presented limited pelvic cancer had much better overall survival than patients who had cancer spreading beyond the pelvic cavity or were diagnosed with NLNM at cancer recurrence.

## Background

Epithelial ovarian cancer (EOC) is one of the most lethal gynecological cancers and patients are usually diagnosed at advanced stages with wide spreading of the cancer. Four routes of cancer spreading in EOC have been identified: by direct extension, by a transperitoneal route, by a lymphatic route, and more rarely, through the blood stream [1].

The lymphatic route of cancer dissemination to retroperitoneal pelvic and para-aortic lymph nodes is rather common in advanced-stage EOC. Affected patients are classified as stage IIIc according to the FIGO staging system. However, a small group of these patients only have retroperitoneal lymph node metastasis without intraperitoneal carcinomatosis and are upstaged from stages I through IIIB disease to stage IIIc. These patients are reported to have a better survival compared with typical EOC patients who show macroscopic peritoneal carcinomatosis [2-6]. Cancer spreading through the lymphatic system could further extend up to the neck lymph nodes. Cases with neck lymph node metastasis (NLNM) are interpreted as distant metastasis and are classified as FIGO stage IV. Based on clinical experience, these cases tend to have poor outcomes; however, cases with NLNM are not common and are rarely reported. To clarify if patients with NLNM could have similar clinical features as patients with retroperitoneal lymph node metastasis, we retrospectively reviewed all EOC patients who were pathologically proven with NLNM in our hospital over a period of 10 years. Furthermore, based on the clinical observation, recurrent ovarian cancers were more chemoresistance and were less effective to current treatment compared with their initial treatments. Therefore, we divided our patients into two groups according to the time of NLNM identification: either at initial exploratory surgery or during cancer recurrence. We evaluated the factors that influenced subsequent disease progression, the pattern of cancer recurrence, and the clinical outcomes, aiming to identify the subgroup of patients with NLNM that might have a better survival outcome.

## Methods

### Patient selection

We reviewed our institution’s medical records to identify all patients with EOC and pathologically proven NLNM between January 2001 and December 2010. Patients with physical neck lymph node enlargement without pathologically proof were excluded from the study. The included patients were divided into two groups. Group A: when NLNM was identified at the same time as initial exploration of the ovarian cancer; Group B: when NLNM was identified at cancer recurrence.

### Treatment and clinical assessment

All patients underwent cytoreductive surgeries by gynecologic oncologists at the Department of Obstetrics and Gynecology as the initial exploration. Optimal debulking surgery was defined as residual tumor with a volume of less than 1 cm. The tumor stage and histological diagnosis of each case was determined according to FIGO criteria and the histological typing system of the World Health Organization, respectively. Chemotherapies based on platinum-containing regimens were performed after surgery. Patients were assessed for clinical response regularly, including physical examination, computer tomography of the abdomen and pelvis, chest x-rays, and testing of CA-125 levels. Clinical progression, an increase in measureable disease, or an increase in CA-125 by 100% was defined as new metastases. Patient characteristics, including age at the diagnosis of NLNM, imaging studies, intra-operative findings, consequences of chemotherapies and surgeries, times and locations of recurrences, and patient outcomes were recorded for each case. All patients were followed-up until they died or until July 2012.

### Statistical analysis

Parametric continuous variables were compared using a *t*-test for independent samples. Non-parametric dichotomous variables were compared using a *χ*^2^ test. Survival time was recorded from the date of NLNM identification to the date of death from disease or date of last seen. Kaplan-Meier analysis with a log-ranking test was used to estimate survival probabilities and compare survival distributions stratified by the time of NLNM identification and by tumor status during operation in Group A patients. Univariate and multivariate regression analyses based on a Cox proportional hazards model were used to evaluate the relative importance of variables as predictors of survival time. The statistical analysis was carried out using the Statistical Analysis System (SAS) version 8.0 (SAS Institute, Cary, NC, USA). Probability values less than 0.05 were regarded as significant.

## Results

### Patient characteristics

Between January 2001 and December 2010, 812 patients with ovarian malignancies were operated in our hospital. Among them, 11 patients identified as having NLNM were classified as Group A; 9 patients identified as having NLNM at ovarian cancer recurrence after previous cancer remission were classified as Group B. All 20 patients received regular follow-up and treatment in our hospital with a zero dropout rate. The clinical features and patient characteristics of these two groups of patients are shown in Tables 1 and 2. The median age of the 20 patients at the time of identification of NLNM was 54.9 years (range: 38–80).

**Table 1:** Clinical features of women with NLNM

**Patient**	**Age at**	**Histology**	**Tumor location**	**Interval between**	**Interval between**	**Recurrence/**	**Survival**	**Dead from disease**
**no.**	**NLNM**		**at initial**	**NLNM and cancer**	**primary surgery**	**metastasis**	**after NLNM**	**at the end of**
	**(years)**		**operation**	**recurrence (months)**	**and NLNM (months)**	**sites**	**(months)**	**follow-up**
Group A								
1	55	Serous	Ovaries	-		-	115	No
2	61	Serous	Ovary	51		LN	82	No
3	48	Serous	Ovary	9		LN	42	No
4	50	Serous	Ovaries and tubes	18		LN	93	No
5	73	Serous	Ovaries and tubes	33		LN	44	Yes
6	43	Serous	Ovaries and pelvic	25		LN	76	No
7	54	Clear	Pelvic and abdominal cavities	Progress		Bone	21	Yes
8	61	Squamous cell	Pelvic and abdominal cavities	Progress			6	Yes
9	46	Serous	Pelvic and abdominal cavities	8		Peritoneal cavity	16	Yes
10	55	Serous	Pelvic and abdominal cavities	15		Brain	18	Yes
11	80	Serous	Pelvic and abdominal cavities, pleural effusion	10		Peritoneal cavity, lung	28	Yes
Group B								
12	66	Serous	Pelvic and abdominal cavities		12	LN	22	No
13	42	Serous	Pelvic and abdominal cavities		13	LN	41	Yes
14	38	Endometrioid	Pelvic cavity		117	LN, peritoneal cavity	6	Yes
15	69	Serous	Pelvic cavity		110	LN, peritoneal cavity, brain	3	Yes
16	44	Serous	Pelvic and abdominal cavities		62	LN, peritoneal cavity, brain	3	Yes
17	59	Clear	Pelvic and abdominal cavities		5	LN, peritoneal cavity, bone	4	Yes
18	48	Clear	Pelvic cavity		20	LN, peritoneal cavity, bone, brain	7	Yes
19	62	Serous	Pelvic and abdominal cavities		13	LN, lung	35	Yes
20	43	Clear	Pelvic cavity		38	LN, brain	1	Yes

LN: Lymph nodes.

**Table 2:** Characteristics of patients with NLNM

	**NLNM at initial surgery**	**NLNM at cancer recurrence**	***P***
	**(Group A) (n = 11)**	**(Group B) (n = 9)**	
Age	56.9 ± 11.3	64.0 ± 11.7	0.19
Parity			
Nulliparous	1 (9.1%)	3 (33.3%)	0.26
Histology			
Serous	9 (81.8%)	5 (55.6%)	0.34
Non-serous	2 (18.2%)	4 (44.4%)	
Tumor size			
≤6 cm	3 (27.3%)	3 (33.3%)	1.00
>6 cm	8 (72.7%)	6 (66.7%)	
CA-125 at operation			
≤35	0	1 (11.1%)	0.35
35–500	5 (45.5%)	2 (22.2%)	
>500	6 (54.5%)	6 (66.7%)	
NLNM after initial surgery			
<1 year		2 (22.2%)	
1–2 years		4 (44.4%)	
>2 years		3 (33.3%)	
Lung or bone metastasis	2 (18.2%)	3* (33.3%)	0.62
Brain metastasis	1 (9.1%)	4* (44.4%)	0.13

* One patient with bone and brain metastasis.

In Group A, 6 patients had tumors confined to the pelvic cavities and 5 patients had cancer spread to the abdominal cavities at initial exploration. Retroperitoneal lymph node dissection was performed in 6 patients and 2 patients received suboptimal debulking surgeries. Chemotherapy was given to all patients following surgery.

In Group B, patients were FIGO staged as Ic (n = 3), IIc (n = 1), and IIIc (n = 5) at the time of initial surgical exploration. Debulking surgeries were optimal, except for 2 patients at stage IIIc and 1 patient at stage IIc. All patients had completed cancer remission after adjuvant chemotherapy. NLNM occurred at a mean period of 43.3 months (range: 5 to 117) after initial surgery.

### NLNM may not occur with pelvic and para-aortic lymph node metastasis

Based on imaging studies, 4 patients in Group A showed no enlargement of pelvic and para-aortic lymph nodes. Among them, only 1 patient received pelvic lymph node dissection and was pathologically proven to be negative. Five patients showed enlargement of pelvic and para-aortic lymph nodes by imaging studies. All patients had pelvic lymph node sampling or dissection; one of them was found pathologically negative. From the 5 patients with image or pathologically negative retroperitoneal nodes, 2 patients had tumors confined to the pelvic cavity and other 3 patients had tumors extended into the abdominal cavity at the time of surgery.

In Group B, all patients received pelvic lymph node dissection at initial exploration, except for one patient who was at stage Ic and showed no enlargement of retroperitoneal lymph nodes. In all patients, the retroperitoneal lymph nodes were pathologically negative with tumors spread to the pelvic cavity and positive with tumors extended into the abdominal cavity. At cancer recurrence with NLNM, 2 patients showed no enlargement of retroperitoneal lymph nodes.

### Clinical outcome

**Figure 1:**
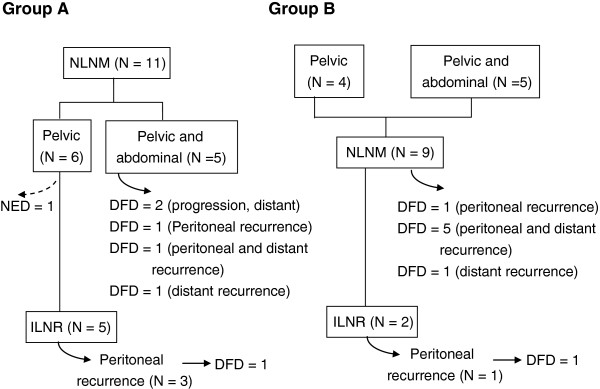
**Flow chart of events according to the occurrence of NLNM, and the location of the tumor during initial surgical exploration.** DFD = dead from disease, NED = no evidence of disease.

The clinical courses of these two groups of patients are delineated in Figure 1.

#### In Group A patients

All patients with tumors that confined to the pelvic cavity showed serous histology. One patient had complete cancer remission 115 months after surgery. Five patients developed isolated lymph node recurrence (ILNR) at a median follow-up of 25 months (range: 9 to 51). Eventually, 3 of them developed intraperitoneal carcinomatosis after a median follow-up of 60 months (range: 42 to 93) and one patient died from the disease 44 months after the operation.

From patients with cancer spread to the abdominal cavity, 2 had non-serous histology; they were chemoresistant and showed cancer progression. Three of the patients had serous histology and had cancer recurrence in the peritoneal and/or distant sites at a mean follow-up of 11 months (range: 8 to 15). All these patients died shortly (within 28 months) after initial surgical treatment.

#### In Group B patients

Two patients showed ILNR and NLNM at 12 and 13 months, respectively, during cancer recurrence. Both patients were at stage IIIc serous histology and received suboptimal debulking surgeries in their initial surgical exploration. Neck masses subsided completely after chemotherapy. However, one of them developed peritoneal carcinomatosis and died 41 months after NLNM identification. The other patient was free of disease 22 months after NLNM identification.

One patient with endometrioid histology and two with serous histology showed repetitive pelvic and/or abdominal recurrence and received multiple debulking surgeries and adjuvant chemotherapy. These patients showed long disease-free periods (37 to 84 months) and NLNM occurred at 62 to 117 months after initial surgery, compared to other patients where NLNM occurred at 5 to 38 months after initial surgery.

Six out of nine patients showed lung and bone metastasis within one month after NLNM, and brain metastasis occurred within six months before or after NLNM. From these patients, four presented with peritoneal cancer spreading (one patient showed peritoneal cancer without distant metastasis) and died within seven months after NLNM. On the other hand, one patient with lung metastasis but no peritoneal cancer survived 35 months after NLNM. Another patient, who presented with clear cell histology, had concurrent NLNM and a stroke at 38 months after her initial surgery; she died 1 month later due to dissemination, intravascular coagulopathy, and sepsis.

### Survival analysis

**Figure 2:**
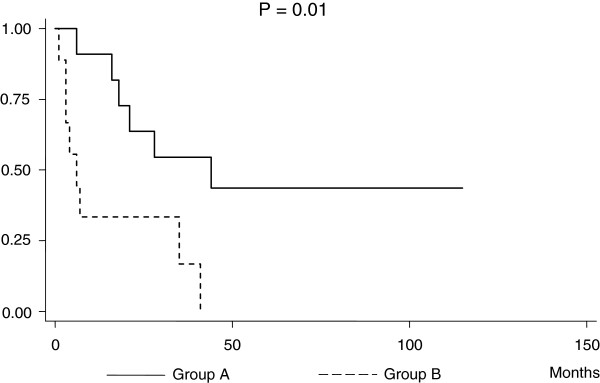
Kaplan-Meier survival estimates of overall survival for patients with EOC stratified by time of NLNM identification.

**Figure 3:**
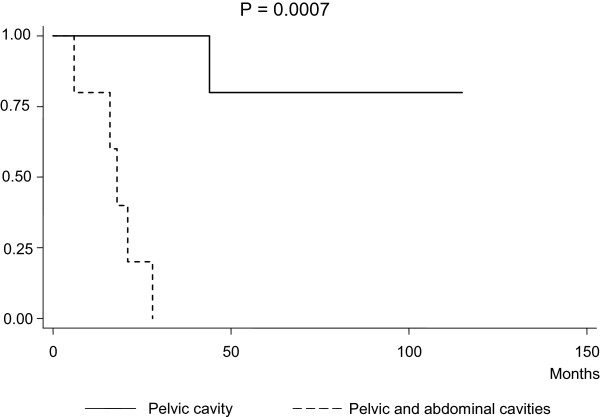
Kaplan-Meier survival estimates of overall survival for patients with NLNM at the time of initial surgery (Group A), stratified by the tumor status during operation.

Figure 2 shows an analysis of survival rates after NLNM in Group A and Group B patients. Patients in Group A survived significantly longer than patients in Groups B (*P* = 0.01). Median survival after NLNM was 42 months (range: 6 to 115) in Group A, but only 6 months (range: 1 to 41) in Group B. Among the patients in Group A, survival time was more prolonged when the tumor was confined to the pelvic cavity than the tumors that had already extended into the abdominal cavity at initial surgical intervention (*P* = 0.0007) (Figure 3). The corresponding median survival periods were 18 (range: 6 to 28) versus 79 (range: 42 to 115) months. Using Cox proportional regression analysis, NLNM identification at the time of initial surgery (Group A) was a more favorable independent predictor for overall survival rates compared with discovering NLNM during cancer recurrence (Group B) (Table 3).

**Table 3:** Survival analysis after diagnosis of NLNM in patients with EOC

	**Univariate Analysis**		**Multivariate Analysis**	
Variables	Hazard Ratio	*P*	Hazard Ratio	*P*
	(95% CI)		(95% CI)	
Age	1.00 (0.95 – 1.05)	0.89	1.06 (0.99 – 1.13)	0.09
NLNM				
At initial surgery (Group A)	1		1	
At cancer recurrence (Group B)	3.91 (1.25 – 12.23)	0.02	4.52 (1.07 – 19.18)	0.04
Histology				
Serous	1		1	
Non-serous	6.90 (1.83 – 26.09)	0.004	18.67 (1.97 – 176.90)	0.01
Bone or lung metastasis	2.21 (0.69 – 7.01)	0.18	0.28 (0.05 – 1.59)	0.15
Brain metastasis	7.76 (2.02 – 29.86)	0.003	6.93 (1.42 – 33.75)	0.02

### Serous histology was associated with better clinical outcome

Histological examination revealed 14 (56%) serous, 4 clear cell, 1 endometrioid, and 1 squamous cell carcinoma (Tables 1 and 2). Based on the Cox proportional hazards model, histological types of EOC proved to be the most significant predictor for overall survival (Table 3). Patients with non-serous histology had an 18.7 higher hazard ratio of death from cancer following NLNM diagnosis compared with patients with serous histology. In Group A, the two patients with non-serous histology (1 with clear cell carcinoma and the other with squamous cell carcinoma) had cancer extension beyond the pelvic region at surgery and were chemoresistant. In Group B, 3 patients with clear cell carcinoma (1 in stage IIIc and 2 in stage Ic at the time of initial surgery) developed distant metastasis (including 2 cases of brain metastasis) at the time of cancer recurrence and expired shortly after the diagnosis of NLNM. One patient with endometrioid carcinoma received multiple surgical explorations and chemotherapies, and despite surviving 117 months before NLNM, died 6 months thereafter.

### Brain metastasis as a late manifestation of NLNM

Nine patients, 2 in Group A and 7 in Group B, developed distant metastasis to the brain, lungs and/or bones. By using a Cox proportional hazards model, brain metastasis was demonstrated to be the second most significant predictor for overall survival with the hazard ratio of 6.93 (*P* = 0.02) (Table 3). Brain metastasis occurred in 1 patient in Group A and 4 patients (44.4%) in Group B. Two of these patients in Group B showed histology of clear cell carcinoma. The mean survival period of these 5 patients was 4.4 months (range: 1 to 10) after the discovery of brain metastasis.

## Discussion

EOC patients with NLNM are classified as FIGO stage IV and are expected to have poor survival outcomes. However, our study revealed a subgroup of EOC patients with NLNM who demonstrated a distinct clinical manifestation with relatively good survival outcomes. These patients received only surgical removal of primary tumors and adjuvant chemotherapy. Although recurrence rates after treatment in these patients were as high those of patients in advanced stages, one patient remained in complete remission for a long period after treatment.

Timing of NLNM identification is a key predictor for patient survival. We found that patients with simultaneous NLNM at initial surgical intervention of primary ovarian lesions had better survival outcomes than patients with NLNM discovered at cancer recurrence. However, among the patients who had NLNM at initial surgical intervention, only those patients who had tumors confined to the pelvic cavity had better survival rates. Patients with ovarian lesions that had spread to the abdominal cavity at NLNM identification had very poor outcomes. Similarly, poor survival rates were observed in EOC patients who had retroperitoneal lymph node metastasis. Patients with retroperitoneal lymph node metastasis without peritoneal carcinomatosis were reported to have an overall survival rate of 58% to 84% compared with 18% to 36% for those with macroscopic peritoneal carcinomatosis, depending on variations in the cancer treatment [2-6]. It could be inferred that NLNM is an extension of retroperitoneal lymph node metastasis and therefore demonstrates similar clinical features and survival outcomes as patients with retroperitoneal lymph node metastasis.

Our findings suggest that there are two distinct biological types of invasive EOC: one is very aggressive and prefers peritoneal invasion, and one that has limited capabilities of peritoneal invasion. In both Groups A and B, some patients appeared to have very aggressive clinical courses at detection of NLNM. They were more likely to be non-serous histology, chemoresistant, presented with early cancer recurrence accompanied with distant metastasis (including brain metastasis), and fatal outcome. However, the exact biological factors that contribute to this unique entity of EOC are unknown and require further research.

On the other hand, a few patients in Group B had ILNR at the time of NLNM. Particularly, all patients in Group A that had tumors confined to the pelvis showed ILNR at the time of cancer recurrence; one of them even showed complete cancer remission. All these patients had serous histology and were good responders to chemotherapy; many of them had complete remission of neck lymph nodes after chemotherapy. Based on the relevant literature, ILNR occurs in about 4% to 6% of patients with EOC [7,8], and represents approximately 10% of overall recurrences [7]. Although it is relatively rare in EOC, it shows amazingly good survival outcomes [7-12]. No obvious predisposing factors have been reported in association with the occurrence of ILNR. We found patients with ILNR were more likely from Group A where EOC tumors were found confined to the pelvic cavity at the time of NLNM. However, after long follow-up periods, peritoneal carcinomatosis eventually occurred in 4 patients, resulting in the death of two patients due to colon obstruction at 41 and 44 months after the discovery of NLNM. These results again support our observation that patients who have confined pelvic tumors during initial NLNM identification belong to a unique EOC group that is biologically less aggressive. Tumors with a limited capability of peritoneal spreading showed ILNR during cancer recurrence. After a longer period, peritoneal carcinogenesis somehow developed and led to patient death. Likewise, Legge et al. observed the occurrence of peritoneal spreading in 25% of their 32 ILNR patients at a median follow-up of 22.5 months (range: 7–96) and subsequent rapid fatal outcomes [7].

Although we observed some commonalities between our NLNM patients and the reported ILNR patients, seven of our patients did not have systemic retroperitoneal lymphatic node metastasis during NLNM. Five of these patients were diagnosed solely by imaging studies, and one patient showed a positive imaging study but pathologically negative retroperitoneal lymph nodes. It has been reported that the diagnostic sensitivity of CT for pelvic and abdominal lymph nodes is low (43%), but the specificity is high (92%) [13]. Even so, the concordance of radiologic staging with surgical staging in ovarian cancer is as high as 78% [14]. However, 15.8% of extra-abdominal lymph node metastasis is unpredictable by PET/CT imaging studies [14]. Therefore, the true incidence of retroperitoneal lymphatic metastasis in our cases could be higher. It is possible that some of our cases might have microscopic retroperitoneal lymphatic metastases that were not detected by radiologic studies or were misdiagnosed without complete or radical lymph node dissection. Even so, lymphatic cancer spreading might not occur systematically along the lymphatic tract. Kleppe et al. reported that in EOC that appeared confined to the pelvis at the time of initial exploration, the mean incidence of lymph node metastases was 14.2% (range: 6.1–29.6%) [15]. In these cases, lymph node metastasis was found to be 4.3% (range 0.0–14.8%) in both the pelvic and para-aortic region, 7.1% (range 3.0–13.0%) only in the para-aortic region, and 2.9% (range 0.0–11.1%) only in the pelvic region [15]. High grade and serous histology were the two risk factors for lymph node metastasis in these apparently early-stages of EOC [16]. This suggested the skipping nature of lymphatic spreading in EOC.

One other limitation of our study was that only enlarged neck lymph nodes that were biopsied and pathologically proven were included. This might reduce the true incidence of NLNM in EOC patients, particularly in cases with neck lymph node enlargement identified during multiple sites of ovarian cancer recurrence (i.e. Group B patients). Our long study period might overcome this limitation.

By survival analysis, non-serous histology of EOC was the strongest predictor for patient survival. All patients with non-serous histology in the two groups had cancer progression or recurrence shortly after treatment. Cancers that regressed completely after treatment were only in patients with serous histology. Brain metastasis was the next strongest predictor for patient survival. Brain metastasis has been reported as uncommon with an incidence of less than 2% in EOC patients [17,18]. The incidence of brain metastasis in our cases was very high. Five patients developed brain metastasis: one in Group A and four in Group B. The high incidence of brain metastasis in NLNM could be explained by the close approximation of the neck lymph nodes to the brain; it also highlights the very aggressive nature of these forms of cancer. Our patients had a mean survival period of 4.4 months after brain metastasis and 2 had clear cell carcinoma histology. Similarly, high mortality rates from brain metastasis have been reported, and brain metastasis are believed to be a late manifestation of ovarian cancer in advanced stages [18].

Several other factors, such as small sample size, varying histology of ovarian cancer, and different causes of death, could have confounded the analysis results. Although NLNM is a rare clinical manifestation in ovarian cancer, the relative small sample size for a retrospective study limits the conclusions. Future studies with larger sample sizes and more rigorous research designs will help to clarify the prognostic value of NLNM.

Our study also suggested that EOC tumors that displayed the lymphatic route of spreading could be more chemosensitive than those with intraperitoneal spreading. In observing that some of our patients with ILNR eventually developed peritoneal carcinomatosis after a long follow-up period, a more aggressive treatment should be considered for patients with ILNR. From literature reports on patients with ILNR, survival outcome was found to be longer in cases that underwent surgical removal of metastatic lymph nodes in addition to chemotherapy [10-12]. When possible, surgical removal of metastatic neck lymph nodes in patients with confined pelvic mass during initial surgical intervention of primary tumor would likely result in a higher chance of complete eradication of the cancer.

## Conclusions

NLNM is a manifestation of the advanced disease status of EOC or a unique entity of EOC that spreads via lymphatic routes. In our analysis, patients with serous histology that was confined to the pelvic cavity at the time of identification of NLNM had relatively better prognosis compared with other stage IV EOC patients. However, these patients were likely to develop ILNR that might eventually lead to intraperitoneal carcinomatosis and result in patient death. Non-serous histology EOC and brain metastasis were the two strongest predictors for poor overall survival in patients with NLNM. The results of our study support the identification of a subgroup of stage IIIc patients whose disease had been elevated from stage I to IIIB based on retroperitoneal lymph node metastases in the pelvic and/or para-aortic lymph nodes, and even to NLNM, without intraperitoneal carcinomatosis.

## Consent

This study was conducted with approval from the institutional review board from the National Taiwan University College of Medicine (201101054RC). Written informed consent was obtained from the patient for the publication of this report and any accompanying images.

## Abbreviations

EOC: Epithelial ovarian cancer; ILNR: Isolated lymph node recurrence; LN: Lymph nodes; NLNM: Neck lymph node metastasis.

## Competing interests

The authors declare that they have no competing interests.

## Authors’ contributions

CWC participated in the data acquisition, data analysis, literature review, and drafted the manuscript of this article. PLT planned the analysis, participated in data acquisition, data analysis, literature review, patient treatment, and drafting and critical revision of the manuscript. CCL participated in data analysis. CAC participated in patient treatment and data analysis. All authors read and approved the final manuscript.
